# Histone Chaperones as Cardinal Players in Development

**DOI:** 10.3389/fcell.2022.767773

**Published:** 2022-04-04

**Authors:** Sruthy Manuraj Rajam, Pallavi Chinnu Varghese, Debasree Dutta

**Affiliations:** ^1^ Regenerative Biology Program, Rajiv Gandhi Centre for Biotechnology (RGCB), Thiruvananthapuram, India; ^2^ Manipal Academy of Higher Education, Manipal, India

**Keywords:** histone chaperone, gametogenesis, fertilization, pre-implantation, post-implantation, gastrulation, organogenesis, pluripotency

## Abstract

Dynamicity and flexibility of the chromatin landscape are critical for most of the DNA-dependent processes to occur. This higher-order packaging of the eukaryotic genome into the chromatin is mediated by histones and associated non-histone proteins that determine the states of chromatin. Histone chaperones- “the guardian of genome stability and epigenetic information” controls the chromatin accessibility by escorting the nucleosomal and non-nucleosomal histones as well as their variants. This distinct group of molecules is involved in all facets of histone metabolism. The selectivity and specificity of histone chaperones to the histones determine the maintenance of the chromatin in an open or closed state. This review highlights the functional implication of the network of histone chaperones in shaping the chromatin function in the development of an organism. Seminal studies have reported embryonic lethality at different stages of embryogenesis upon perturbation of some of the chaperones, suggesting their essentiality in development. We hereby epitomize facts and functions that emphasize the relevance of histone chaperones in orchestrating different embryonic developmental stages starting from gametogenesis to organogenesis in multicellular organisms.

## Introduction

Chromatin organization and its dynamicity likely regulate the DNA-templated processes including DNA replication, DNA repair, and gene expression (Groth et al., 2007). The maintenance of genomic and epigenomic integrity and functionality is controlled by the regulated assembly and disassembly of nucleosomes (Hammond et al., 2017). The nucleosome is the basic unit of chromatin consisting of 147 bp DNA along with linker DNA and linker histone H1 wrapped around histone octamer having two copies of core histone H2A-H2B and H3-H4. Tetramer of (H3-H4)_2_ flanked by two dimers formed by H2A-H2B. The conserved histone families exist as variants that are amenable to several post-translational modifications and are differentially expressed. The process of nucleosome assembly and disassembly accomplishes in a step-wise fashion. The old histones/histone variants are replaced or the newly synthesized histones are deposited onto the newly synthesized DNA during the cell cycle. The factors including ATP- dependent chromatin remodeling complexes, non-histone chromatin-associated proteins, post-translational modification enzymes, and histones chaperones facilitate nucleosome assembly and disassembly by histone transport, deposition, eviction, and storage. Histones and their variants are escorted by distinct histone chaperones in most of the events of histone metabolism, right from its synthesis to recruitment into the chromatin for nucleosome assembly, as well as disassembly and eviction. Mechanically, histone chaperones function by shielding histone interfaces and trapping in non-nucleosome conformations (Hammond et al., 2017). They are often found interacting with the nucleosome during the key cellular processes. The histone molecules along with the escort molecule, histone chaperone, and the modifications in different combinations generate a unique pattern to determine the cell fate by maintaining the chromatin in an open or closed state. The loss of the chaperoning function has been reported to affect genomic stability, cell cycle arrest, DNA damage, or cell death. This higher-order packaging of the eukaryotic genome into the chromatin, mediated by histones and associated non-histone proteins determines the states of chromatin during processes related to DNA metabolism. The exchange and turnover of histone during chromatin reorganization directly or indirectly alter the expression of various genes. In addition, it is noteworthy that chromatin structure varies across the genome. It depends on whether the gene is being actively transcribed or the location is within the exon, intron, or other regulatory elements. The chromosome location also should be considered, as the chromatin near the centromere or telomere is considerably different from other regions of a chromosome. The variations are even observed across different cell types as the composition of chromatin from germline substantially differs from that of somatic, transformed, and embryonic stem cells.

The development of a multicellular organism begins with gametogenesis followed by fertilization to embryogenesis and morphogenesis to become an adult. Once the gametes are produced, the male and female gametes come together and fuse either by internal fertilization or external fertilization. In mammals, the events of fertilization followed by the zygotic genome activation (ZGA), new zygotic information activated by maternal program, coincide with the pre and post-implantation embryogenesis. Each transition event of pre and post-implantation is of utmost critical importance in determining the successful completion of pregnancy. The developmental transition stages are marked by the dramatic alteration of the chromatin architecture particularly in the events of fertilization, compaction, blastocyst formation, gastrulation, and organogenesis. These alterations are brought about by the interplay of the transcription factors and epigenetic modifiers. In this review, we aim at providing an insight into the significance of histone metabolism mediated by histone chaperones in the maintenance of the chromatin state of different genes or loci during development. The road from start to finish has been explored here. Histone chaperones right from the initiation of gamete formation to their fertilization followed by extension to pre- and post-implantation stages, propagation to gastrulation stage and finally to the decisive states of differentiation along with the regulation of pluripotency that forms the basis of the cells to differentiate, have been discussed in detail.

### Gametogenesis

Extensive remodeling and reorganization of the genome mark the development of highly specialized male and female germ cells. The major chromatin-templated event during gametogenesis includes the condensation of germ cell chromatin, reestablishment of genomic imprints, and the incorporation of certain histone variants and post-translational modifications in the genome to distinguish autosomes and sex chromosomes. Gametogenesis is the formation of gametes that include both the production of sperms (spermatogenesis) in males and eggs (oogenesis) in females through the process of meiosis. Meiosis ensures the haploidization and the independent assortment of the genome within the gametes.

#### Spermatogenesis

**FIGURE 1:**
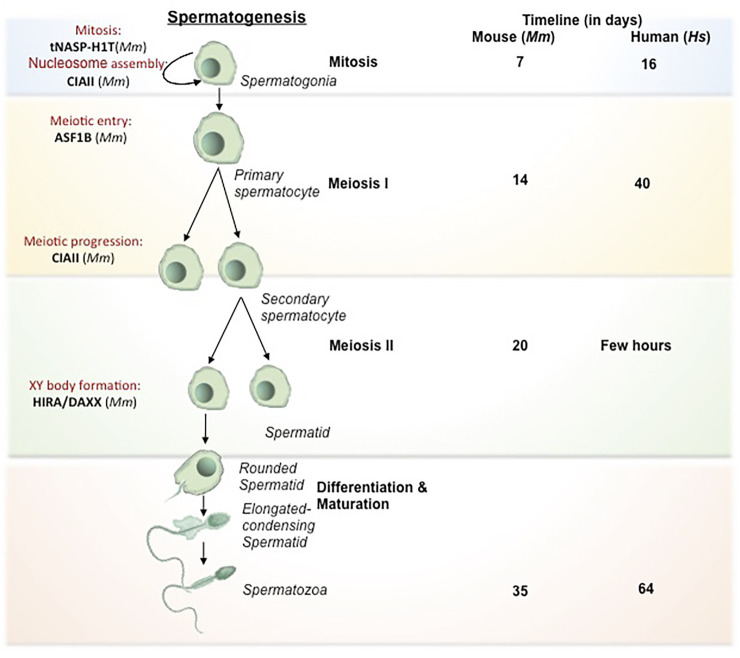
Histone chaperones in different stages of spermatogenesis. Schematic representation of spermatogenesis along with the timeline has been depicted. The transformation from germ cells to gametes is subdivided into 4 stages based on the key processes involved viz. mitosis (self renewal of germ cells), meiosis I (reduction division) meiosis II (equational division similar to mitosis) and differentiation/maturation (transformation to functional, fertilization competent spermtaozoa). The functional implication of different histone chaperones in the corresponding stages of spermatogenesis is mentioned. NASP and CIAII are involved in the self renewal of spermatogonial cells. ASF1B takes part in the regulation of mitotic to meiotic shift while CIAII regulates the meiotic progression in the spermatocyte. HIRA and DAXX are involved in the XY body formation in the pachytene spermatocytes just before the entry to the differentiation. During the spermiogenesis, the differentiation phase of the mature spermatids to the spermatozoa, the protamine loading is mediated by CAF1 and NASP. Abbreviation-Mm, *Mus musculus*.

The development of male germ cell occurs in three phases, which includes the self-renewal of spermatogonial cells by mitosis followed by the reduction division of spermatocytes to form spermatids and subsequent transformation to spermatozoa by spermiogenesis (Yao et al., 2015). A schematic representation of spermatogenesis along with the timeline has been mentioned in Figure 1. The entire process of spermatogenesis is completed in around 35 days in mice whereas it takes almost 65 days in human. The spermatogonial stem cells (SSCs) arise from primordial germ cells (PGCs) that mark the initial stage of male germ cell formation. The committed SSCs undergo meiosis to form the haploid spermatocytes (Manku and Culty, 2015). The SSCs undergoes continuous mitosis to replenish its pool in the testis and to generate the progenitor populations for the generation of sperm. Any abnormalities in the self-renewal and progenitor generation, ultimately leads to failure in spermatogenesis. The linker chaperone, Testis-specific nuclear autoantigenic sperm protein (tNASP) is indispensible for cell cycle regulation in spermatogonial cells. The higher expression of linker histone H1T coincides with the tNASP expression in proliferating spermatogonial cells in mice. The association of tNASP-H1T complex with the Heat Shock Protein 90 Alpha Family Class A Member 1(HSP90AA1) facilitates the translocation of cytoplasmic NASP to the nucleus (Alekseev et al., 2005) where it associates with Chromatin Assembly Factor 1A subunit (CHAF1A) in a multi-chaperone complex for G1/S progression in spermatogonial cells (Groth et al., 2005). In human and mouse, another H3/H4 chaperone, CCG1 interacting Factor II (CIA-II) was identified to be profoundly expressed in the testis and proliferating cells. However in mice, mCIAII expression is restricted to pre-meiotic and meiotic germ cells. It is proposed that this chaperone could possibly be involved in the recruitment of testis-specific histones for the nucleosome assembly in spermatogonial cells (Umehara and Horikoshi, 2003). Though, the presence of CIAII is evident in male germ cells, its distinctive function in spermatogonial cell remain to be explored.

The next phase is the commitment of male germ cells to meiosis, which is critical for the generation of spermatozoa in the testis. Growth factors and hormones are responsible for the initiation and tight regulation of meiosis in cells, which are committed to spermatogenic lineage. The replicative histone H3 (H3.1 and H3.2), the non-replicative variants H3.3 and its chaperones are involved in regulation of different events of spermatogenesis. The incorporation of testis specific H3 variant, H3T allows germ cells to enter meiosis by providing the open chromatin structure (Ueda et al., 2017). Mammalian H3.3 have two isoforms H3.3A and H3.3B, which are expressed until the pre-meiotic and meiotic-prophase spermatocytes respectively (Bramlage et al., 1997). In mice, the perturbation of H3.3A and H3.3B impairs male fertility (Couldrey et al., 1999; Hödl and Basler, 2009; Bush et al., 2013; Tang et al., 2015). The increase in expression of H3.3 variant in the testis coincides with the expression of the corresponding chaperones. This observation is in agreement with the higher expression of H3.3 chaperone, Anti-silencing Factor B (ASF1B) in the proliferative compartment of progenitor cells. Upregulation of ASF1B in male germ cell directly or indirectly enhances the transcriptional activation of *Stra8* (Stimulated by Retinoic Acid) gene, which regulates the meiotic entry and mitotic/meiotic shift. Retinoic acid and STRAT8 signaling primarily regulate the mitotic to meiotic shift (Anderson et al., 2008). Hence, the loss of ASF1B function in mice results in delayed meiotic entry (Messiaen et al., 2016). Despite being reproductively compromised, *Asf1b* knockout mice are viable.

Meiosis begins with the pairing of homologous chromosomes, which is followed by the formation of synapse for the exchange of genetic material by crossing over and recombination. Recombination is critical for the establishment of synapse between the homologous chromosomes for the exchange of paternal alleles. This event take place during prophase I which is divided into sub-stages: leptotene, zygotene, pachytene and diplotene. The synapse formation completes at pachytene stage. The chromosomes are prone to meiotic errors during the cross over and recombination. The meiotic check point thereby ensures the fidelity of the chromosomes and the DNA damage repair and this surveillance mechanism is active in the pachytene spermatocytes. The synpased region involves in the recombination whereas the unsynapsed region becomes transcriptionally silenced. The silencing of unsynapsed region prevents the error-prone crossing over events by the process called meiotic silencing of unpaired chromosomes (Kelly and Aramayo, 2007). Most likely, this event affects X and Y-chromosomes as they have the least homology. In consequence, they form XY body. The histone H3.3 chaperones, Histone Cell Cycle Regulator A (HIRA) and Death Domain Associated Protein (DAXX) are detected in the XY body. Both the chaperones are reported to be involved in the global H3.3 incorporation at the time of Meiotic sex chromosome inactivation (MSCI) in late pachytene spermatocytes for the formation of XY body (van der heijden et al., 2007; Rogers et al., 2004; Szenker et al., 2011). MSCI refers to the transcriptional silencing of the genes in the X and Y-chromosomes followed by the condensation of the chromatin for the XY body formation. MSCI is critical for the meiosis to proceed. Any impairment in this process leads to meiotic arrest at pachytene stage and apoptosis of spermatogonial cells (Handel, 2004). The histone variants H2A.X and macroH2A1.2 are associated with the chromatin condensation in XY body in mice (Fernandez- Capetillo et al., 2003). For the reorganization of synapsed chromosomes in the pachytene spermatocyte, the histone variants and their respective chaperones participates in the nucleosome assembly and DNA repair events. The linker histone H1T present in the pachytene spermatocytes are incorporated throughout the chromosomes (van der Heijden et al., 2007). tNASP-H1T has been found to be associated with the synaptonemal complex in the pachytene spermatocytes for the meiotic progression. The synaptonemal complex is a protein structure that is formed between the two homologous chromosomes during meiosis I in eukaryotes. It is mediated through synapsis and recombination and is absolutely critical for the crossover. Heat Shock protein Family A (Hsp70) Member 2 (HSPA2) is a lateral component of synaptonemal complex, which provides a link between the synapsed chromosome and the cell cycle in the pachytene spermatocyte. Interaction of tNASP-H1T with HSPA2 enhances its ATPase activity and facilitates the translocation of the HSPA2-tNASP-H1T complex to synaptonemal complex. There it associates with the cell cycle regulator Cyclin Dependent kinase 1 (CDK1/CDC2), which is required for G2 to M transition in prophase I (Alekseev et al., 2009).

Following meiosis, the spermatids undergo morphogenesis to form spermatozoa by the process called spermiogenesis. The initial remodeling of the spermatid genome is established by the genome-wide histone H4 hyperacetylation (Meistrich et al., 1992) to facilitate the open chromatin configuration for the subsequent displacement of somatic histones. The first phase of the process is marked by the conversion of histone-based chromatin to highly specialized testis-specific arginine-rich protamine based chromatin (Goldberg et al., 1977), which is a unique feature in eukaryotes. The second phase is the crosslinking of protamine by the formation of disulphide bonds in late spermatid for the exit (Golan et al., 1996). The protamine-associated genome contributes to the hydrodynamicity and condensation of chromatin and facilitates its packaging into the nucleus during sperm head formation in spermiogenesis. The histones are evicted from the nucleosome concomitant with the incorporation of transition proteins (TNPs) followed by its replacement by protamines. The histone H4 hyperacetylation in elongating spermatids establishes the open chromatin configuration for the histone displacement. The reduced level of H4 acetylation causes impaired fertility in humans and mice (Sonnack et al., 2002; Fenic et al., 2004). The canonical histones (core histones and linker histones) and the somatic histone variants are replaced by testis-specific histone variants. H3T is testis-specific and synthesized in spermatogonia and is expressed in early spermatids (Trostle-Weige et al., 1984). The reciprocal expression of testis-specific histone variant TH2A/TH2B controls the histone–nucleoprotamine transition during spermiogeneisis. TH2A gradually declines by the condensation of spermatid nucleus while TH2B replaces the canonical H2B in condensing spermatid (Montellier et al., 2013; Shinagawa et al., 2015). In mice, double knockout of *Th2a*/*Th2b* result in sterile male with very few sperms in epididymis (Shinagawa et al., 2015). However, the *Th2b* null mice show normal spermiogenesis and fertility suggesting that the double knockout phenotype is probably contributed either by *Th2a* alone or the synergistic effect with *Th2b*. The linker histones and their variants expressed in male germs cells are also critical in the preparation of the chromatin structure for histone to protamine transition. In *D. melanogaster*, CAF1-P180/P75 subunits and tNASP is associated with the histone-protamine exchange during the sperm chromatin assembly (Rathke et al., 2010; Doyen et al., 2015). The protamine based toroidal chromatin structure is different from the usual somatic nucleosomal array and contributes to increased compaction of the sperm chromatin making it inaccessible for replication or transcription (Doyen et al., 2013).

Due to the easy accessibility and limited ethical concern of different model system, development studies are mostly performed in lower mammals and hence the discussed content hereby include mouse and few lower organisms as the model system. But an interesting study on male infertility in human, revealed that deregulation of many DNA repair and apoptosis modulating genes associate with human male infertility (Cheung et al., 2019). Among them, Aprataxin PNK Like Factor (APLF), a core histone and macroH2A chaperone having DNA repair activity is remarkably under expressed in infertile males suggesting its essentiality in male fertility (Cheung et al., 2019). However, the mechanism by which APLF regulates male fertility remains elusive.

It is evident from the studies, performed majorly in mice, that an intricate network of histone chaperones, histone variants and histone modification guides the compaction and relaxation of sperm chromatin at different stages of spermatogenesis thereby guiding and regulating the entire process (Figure 1).

#### Oogenesis

**FIGURE 2:**
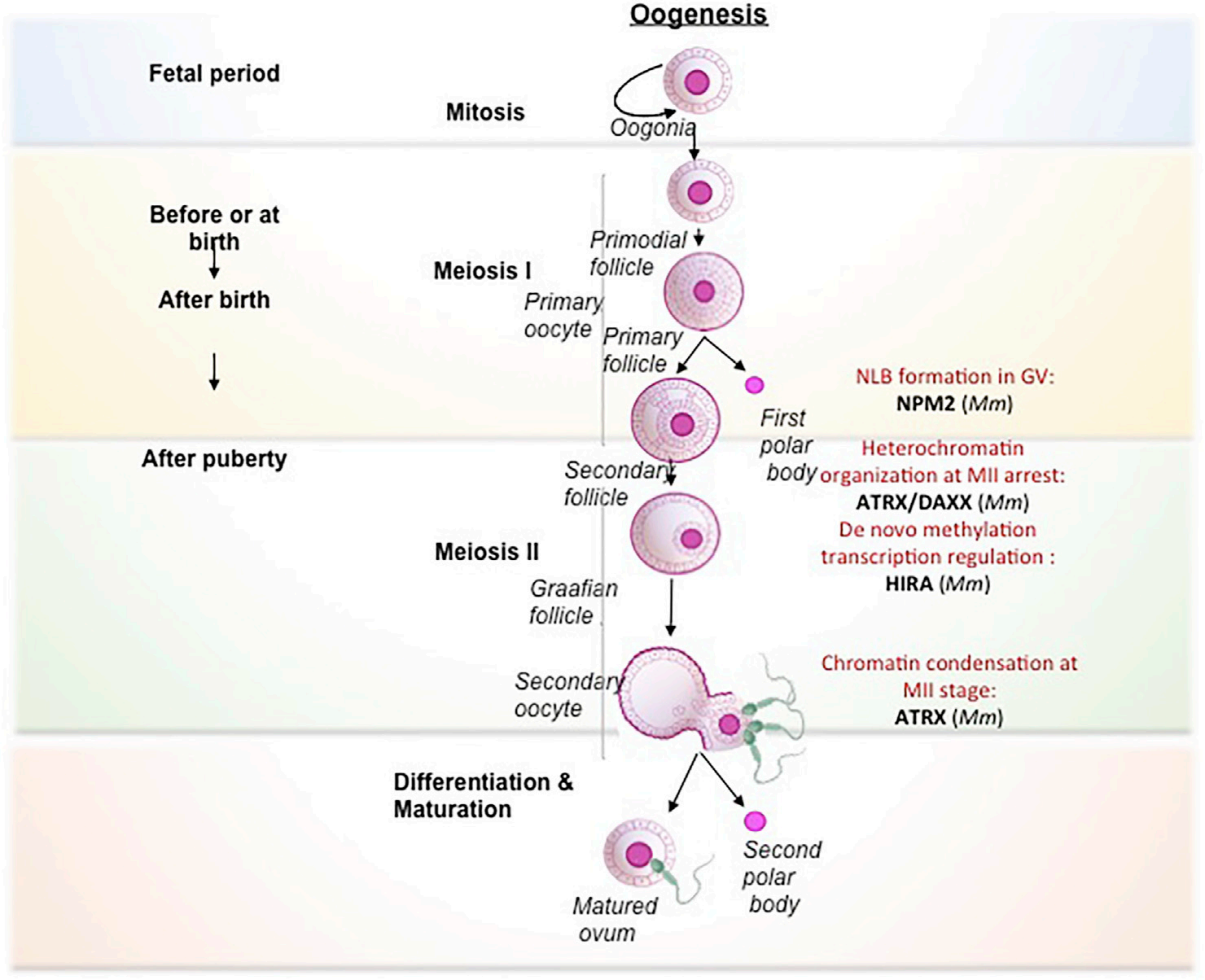
Histone chaperones in different stages of oogenesis. Schematic representation of oogenesis has been depicted. The transformation from oogonia to ovum is subdivided into mitosis (self renewal of oogonial cells), meiosis I (reduction division), meiosis II (equational division similar to mitosis) and maturation (transformation to fertilization competent ovum). The primary oocyte gets arrested at Prophase I of Meiosis I in the fetal period and completes at the time of ovulation. Following that, the primary o/ocyte undergoes Meiosis II and gets arrested at Metaphase. The secondary oocyte thus formed resume to complete meiosis II only upon fertilization to form fully matured ovum. The functional implication of different histone chaperones in the corresponding stages of oogenesis is mentioned. The self renewal of oogonial cell is regulated by CAF1. NPM2 involves in the cortical granule formation and the NLB formation in the primary follicle. NAP1 deposits the B4 linker histone during meiosis I. The H3.3 chaperones, HIRA and ATRXX/DAXX involve in the heterochromatin formation and transcriptional regulation respectively while H3.1/H3.2 chaperone, CAF1 facilitates the chromatin assembly. NPM2 involves in the regulation of oocyte competency for fertilization during the maturation phase. Abbreviation-Mm, *Mus musculus*.

The process of oogenesis involves the development of oocytes from PGCs by meiotic recombination and genome reorganization followed by its activation at the time of fertilization (Figure 2). PGCs proliferate in the ovary by mitosis to form the oogonia, which then subsequently develop into primary oocytes. The oogonia undergoes first meiosis and gets arrested at diplotene stage of prophase 1, lasts until puberty in human whereas in lower organisms like *Xenopus*, it lasts for over a year (Carlson, 1996), and completes only before ovulation. During the arrest, the folliculogenesis event is initiated resulting in the formation of primary follicle. The resumption of meiosis I lead to the breakdown of nuclear envelope in the germinal vesicle (GV) and formation of secondary oocyte and release of the first polar body. The secondary oocytes thus formed goes through second meiotic division and progresses only to metaphase II and get arrested. Upon fertilization, secondary oocyte completes meiosis II division, retaining all the maternal cytoplasm for the initial development of the embryo. The fertilization competency in oocyte is established through global transcriptional changes and *de novo* DNA methylation. The onset of methylation marks coincide with the primary to secondary oocyte transition and is completed at GV stage, when the oocyte becomes transcriptionally inert (Tomizawa et al., 2012).

In mammals, primordial oogonial cells have less abundant oocyte specific-linker histones and the expression is remarkably increased in primary oocyte substantiating its vital role in mitotic to meiotic transition (Furuya et al., 2007; Pan and Fan, 2016). The replacement of histones by the oocyte-specific histone variants is critically important for transcriptional regulation and oocyte reprogramming (Nashun et al., 2015). Histone variant H3.3 is required for female fertility (Hödl and Basler, 2009; Sakai et al., 2009). Similar to spermatogenesis, continuous H3.3–H4 turnover and the expression of HIRA are critical for the transcriptional regulation of genes for developmental progression. Mouse primordial oocyte-specific knockout of *Hira* results in a significant increase in oocyte death and aberrant chromosome structure due to increased DNA accessibility, segregation defects, and responsiveness to DNA damage upon reduced H3 and H4 replacement (Nashun et al., 2015). Continuous reduction in H3.3/H4 level in consequence of HIRA depletion leads to aberrant transcriptional profile and increased sensitivity to DNaseI. This results in the accumulation of more DNA damage as evidenced by an increased level of γ-H2AX and upregulation of DNA-responsive genes. Additionally, *de novo* methylation are drastically reduced in HIRA-depleted oocytes. Methylation at CpG islands are greatly reduced in *Hira* depleted oocytes (Nashun et al., 2015). The H3.3 deposition in oocyte chromatin cannot be compensated by other H3.3 histone chaperones including ATRX/DAXX. Basically, *Hira* mutant oocytes fail to undergo cleavage to 2-cell stage embryo when developed parthenogenetically (Lin et al., 2014).

The second meiotic arrest occurs at the metaphase II in the pre-ovulatory oocyte. The nuclei-germinal vesicle (GV) displays a unique chromatin configuration to confer the oocye with meiotic potential and fertilization competence. GV oocytes become transcriptionally inert by global transcriptional repression and methylation. During this time, the oocytes rely on the stored maternal factors for sustaining the meiotic resumption and further stages until pre-implantation. GV oocyte is characterized by the nuclei condensation and formation of nucleolus like body (NLB). These specialized structures reassemble into pronuclei and form nucleolus bodies after fertilization. Nucleophosmin 2 (NPM2) is involved in the formation of NLB in GV oocytes in mouse. *Npm2* knock out oocyte demonstrated aberrant heterochromatin formation outside nucleolus and exhibited a reduction in clearing acetylated nuclear H3. These mice are viable but fertility is compromised in females. In *Xenopus* and *Zebrafish*, the transcript variants, *Npm2, Npm2a*, and *Npm2b* are expressed predominantly in early oocytes and they take part in cortical granule (CG) formation during folliculogenesis. NPM2 deficiency impairs the egg activation in stage II follicle (Cheung et al., 2018), but the mechanism by which NPM2 affects the oocyte competence is yet to be deciphered. Similar to the *Npm2* knockout, loss in fertility along with impaired gonad development has been observed in *Asf1b* female knock out mice (Messiaen et al., 2016). ASF1B regulate the process of meiotic entry of oocytes and hence its loss results in altered oocyte pool and finally affect fertility in the female knockout mice.

The heterochromatin composition at the chromocentre of preovulatory oocytes is preserved by ATRX/DAXX complex (Baumann et al., 2010). ATRX recruits transcriptional regulator DAXX at pericentric heterochromatin in the pre-ovulatory oocytes. The loss of maternal function of ATRX at the metaphase II stage affects female fertility presumably by impairing the chromosome modification, condensation, and segregation of chromosomes. Lack of ATRX function in matured MII oocyte resulted in misaligned chromosomes, chromosome lagging and in some instances led to the premature anaphase onset with the formation of chromosome bridge. Consistent with this observation, ATRX ablated oocytes exhibited reduced level of H3 phosphorylation (H3S10) at pericentric heterochromatin region, which is responsible for proper condensation and chromatin cohesion and centromere stability during meiosis II. Additionally, maternal ATRX ablation affects the transmission of aneuploidy and genomic stability in oocytes (Baumann et al., 2010). Upon sperm entry, the MII arrested oocyte completes the meiosis II and gets activated for the preparation of embryo development.

Interplay of epigenetic factors of histone chaperones and histone variants significantly contribute to the condensation or decondensation of oocyte chromatin thereby regulating not only the different stages of oogenesis (Figure 2) but also towards the competency of the oocyte for the next stage of fertilization. Gametogenesis sets the tone for the union of the gametes to form the zygote and hence the first stage of development.

### Fertilization

The event of fertilization begins with the entry of spermatozoa into the oocyte followed by the formation of male and female pronuclei and its fusion to form the zygote. At this phase, the oocyte resumes and completes the second division of meiosis and eliminates the second polar body leading to its activation and formation of female pronuclei. On the other hand, the sperm decondenses and assembles into the nuclear envelope to form the male pronuclei. Despite the union, both female and male chromosomes remains separated as pronuclei, they undergo chromatin decondensation and extensive remodelling. In fact, the male pronucleus undergoes extensive remodeling in comparison to the female counterpart. Perhaps, these chromatin modifications are pre-requisite for the acquisition of totipotency, which refer to the potential of cells to differentiate into all three lineages as well as the maternal-fetal connections. The sperm-specific protamines are replaced by maternally supplied canonical/non-canonical histones substantiating the fact that gamete-specific variants are replaced by the somatic-variants to establish *de novo* nucleosome assembly (Nonchev and Tsanev, 1990; van der Heijden et al., 2007). Interestingly, during fertilization, the genomes of sperm and the oocyte are transcriptionally repressed. During this period, the embryo sustains by utilizing the maternally derived factors for the pronuclei formation and the maternal to zygotic transition (MZT) (Li et al., 2013). De-repression of chromatin-mediated repression of the paternal and maternal genome initiates the zygotic gene expression, which allows the transition from germ cells to totipotent embryos. The zygotic gene expression happens first in the paternal genome at the one-cell stage. However, ZGA in the maternal genome occurs at the 2-cell stage embryo (Li et al., 2013).

The sperm chromatin decondensation process involves two steps: the eviction of protamines and the *de novo* assembly of the chromatin by the histones. The extrusion of protamines is considered to be the initial step of paternal chromatin remodeling which happens before decondensation of sperm chromatin (McLay and Clarke, 2003). In mice and *Drosophila*, sperm protamine removal and histone deposition are mutually exclusive events and is regulated by Nucleophosmin (NPM), but in *Xenopus*, the protamine removal is independent of histone deposition mediated by NPM. Of note, NPM2 acts as a protamine acceptor for sperm chromatin decondensation in the NLB of fertilized oocyte in mouse (Inoue et al., 2011). In *Drosophila*, NAP1 and NPM are indispensable for the eviction of protamine A from the sperm chromatin (Emelyanov, et al., 2014).

*De novo* nucleosome assembly at the time of fertilization is not accompanied by DNA synthesis. The nucleosome assembly process is of utmost critical during paternal chromatin remodeling as it governs the subsequent nuclear events including nuclear pore complex (NPC) assembly and nuclear envelope formation. The nucleosome-depleted paternal pronuclei prevent the enrichment of AT-hook Containing Transcription Factor 1 to the nuclear rim and result in the non-functional pronucleus surrounded by a nuclear envelope devoid of NPCs, that impairs the nuclear import and nuclear expansion (Inoue and Zhang, 2014). The deposition of H3.3-H4 onto the paternal chromatin is considered the first step to establish the *de novo* nucleosome assembly during pre-implantation development (Inoue and Zhang, 2014; Nashun et al., 2015). Apparently, the deposition of histone H3 onto the paternal chromatin is in the form of its variant H3.3 along with H4 instead of H3-H4 initially. Maternally supplied H3.3 is globally deposited in the paternal chromatin before the onset of ZGA (Torres-Padilla et al., 2006). The deposition of H3.3-H4 is mediated by the histone chaperone HIRA (Loppin et al., 2005; Lin et al., 2013; Lin et al., 2014). HIRA is critical for the replication-independent (RI) nucleosome assembly pathway that is specifically involved in the deposition and eviction of histone variant H3.3. In mammals and in *Drosophila*, the HIRA complex is critically important for the *de novo* nucleosome assembly on the paternal chromatin (Loppin et al., 2005; Orsi et al., 2013; Lin et al., 2014). HIRA does not contribute to the initial stages of paternal genome reprogramming involving the removal of protamines, however, it is essential for the incorporation of H3.3 within the decondensed sperm chromatin along with acetylated H4. In *Hira* mutant zygotes, both paternal and maternal pronuclei display significant reduction in DNA replication and transcription (Lin et al., 2014). The H3.3/HIRA axis contributes to the transcription of rRNA that is indispensable for the first zygotic cleavage (Lin et al., 2014). In mice, HIRA is also responsible for the deposition of histone H3.3-H4 dimer onto the paternal chromatin and thus co-localizes with the expanding sperm chromatin in the one-cell stage (van der Heijden et al., 2005). In agreement with this observation, a recent study has been reported that the loss of function of maternal HIRA complex (HIRA, UBN1, and CABIN1) in mice fails to properly form the male pronuclei and results in the formation of zygote with one pronucleus (1PN) instead of 2PN condition. The overexpression of HIRA could partially rescue the abnormal male pronucleus formation phenotype. In coherence with HIRA mutant phenotype in mice, the human 1PN zygote observed to have a loss of function of the HIRA complex. The underlying mechanism is unknown. However, they envisaged the likelihood of HIRA-mediated H3.3 incorporation mechanism and the possibility of HIRA to associate with factors that phosphorylates protamine for paternal reprogramming (Smith et al., 2021). In *Drosophila*, after the eviction of the sperm nuclear basic proteins (SNBPs), the HIRA core complex which includes HIRA and Yemanuclein (YEM), fly ortholog of mammalian Ubinuclein 1 (UBN1), localizes in the decondensed sperm chromatin allowing the deposition of H3.3–H4 in the form of (H3.3–H4)_2_ tetramer (Orsi et al., 2013).

The deposition of H3.3-H4 is found to be a pre-requisite for the incorporation of H2A-H2B dimer as the nucleosome assembly is completely abrogated in the absence of either H3.3 or HIRA. In mammals and *X. levis*, H2A.X, a highly conserved variant of histone H2A is incorporated in the sperm chromatin (Ohsumi and Katagiri, 1991; Dimitrov et al., 1994; Nashun et al., 2010). H2AX incorporation is lower in female pronuclei as compared to male, possibly due to the gradual replacement of H2A.Z and macroH2A by H2A.X from the female genome (Chang et al., 2005). In *X. laevis*, NPM3 involves the deposition of H2A/H2B in the paternal pronuclei. NAP1 also deposits H2A/H2B variants for the *de novo* nucleosome assembly.

Soon after the fertilization, the zygote undergoes several cleavages that give rise to a set of cells with distinct characters. The zygote loses the totipotent character while it gains the pluripotent nature.

### Pre and Post-Implantation Embryogenesis

Following fertilization, the reorganized maternal genome reforms by ZGA for priming the embryo towards subsequent developmental stages. In mammals, the zygote undergoes mitotic cleavage divisions, and the blastomeres further compacted to form blastocyst for implantation. Post implantation, embryos transform to specify the cell types and lineages and enter into certain differentiation events.

In mice, maternal HIRA is required for the development of zygote (Lin et al., 2014). *Hira* mutant zygotes are arrested at the 2-cell stage during development. Mechanistic analysis has revealed that maternal HIRA incorporate histone variant H3.3 within the nucleolar precursor bodies and thereby regulate RNA Pol I transcription for ribosomal RNA synthesis which is turn is required for RNA biogenesis at the female pronucleus (Lin et al., 2014).

The reorganization of heterochromatin is critically important for developmental transition stages in pre-implantation embryos. The structural alteration of pericentric heterochromatin from ring structure to dot-like chromocenter is one of the major events in zygotic to 2-cell stage transition in mammals (Probst et al., 2010). The maternal factor, STELLA regulates the expression of H3.3 chaperone DAXX in mediating chromocenter formation in 2-cell stage embryos. The impairment in chromocenter formation in *Stella* null oocyte can be fully compensated by DAXX mediated H3.3 incorporation and the expression of major satellite repeats (Arakawa et al., 2015). The zygotic to further developmental transition stages are enabled by the activation of the embryonic genome post-fertilization. Maternally derived transcription elongation factor SPT6, an H3-H4 chaperone interacts with chromatin remodeler ISWI, that recruits the lysine methyltransferase SET Domain Protein 2 (SETD2) on to the chromatin to regulate H3K36me3 through AKT signaling and is essential for mouse genome activation (Oqani et al., 2019). *Caf1* knockout mice get arrested during or at the start of the compaction stage (8–16 cell stage) of pre-implantation development (Houlard et al., 2006). This is due to the defects in heterochromatin organization and repression of transposable elements in knockout mice during the pre-implantation stage, most likely due to improper distribution of H3 variants. The homozygous mutant of *Caf1p15*0 exhibits embryonic lethality at the stage of compaction (16 cell stage) due to changes in the constitutive heterochromatin organization. The organization of heterochromatin in *Caf1p150* mutant 16 cell embryos resembles 2 to 4-cell embryos, which substantiates the fact that CAF1 is indispensable for the maturation of heterochromatin during pre-implantation stage (Houlard et al., 2006). The developmental transition from two cell to 8 cell stage is marked by global demethylation due to the restricted mobility of DNA Methyltransferase 1 (DNMT1) and low expression of DNA Methyltransferase 3a/3b (DNMT3a/3b) (Cirio et al., 2008). Surprisingly, a study has reported the role of H3-H4 chaperone complex ATRX/DAXX in silencing the repetitive elements by the recruitment of SUV39H leading to H3K9 trimethylation, when DNA methylation levels are low in pre-implantation embryo and mouse embryonic stem cells (ESCs) (He et al., 2015). CAF1 protects the hypomethylated pre-implantation mouse embryos from retrotransposons by repressive histone marks (H4K20me3 or H3K9me3) (Hatanaka Y et al., 2015). *Spt6* null embryos fail to compact to morula possibly as a consequence of defects in mRNA processing. In mice, knockout of either *Atrx* or *Daxx* or *Caf1* causes embryonic lethality (Michaelson et al., 1999; Garrick et al., 2006). The loss of ATRX leads to the transmission of aneuploidy and centromere breaks resulting in structural and numerical aberrations in the pre-implantation embryo (Baumann et al., 2010). The male *Atrx* null embryos do not survive beyond 9.5 d.p.c (days postcoitum) due to defective extra-embryonic trophoblast formation, the first differentiated lineage in developing embryos (Garrick et al., 2006). One of the less explored core histone chaperones, APLF has been recently reported as a crucial factor required for implantation of mouse embryo (Varghese et al., 2021). APLF is predominantly expressed in the trophectoderm (TE) of the blastocyst and the lineages derived from TE. *Aplf* knockdown embryos experienced early hatching *in vitro* and failed to implant *in vivo* (Varghese et al., 2021). The mechanism for regulation is not clear to date but the role of APLF in regulating the epithelial-to-mesenchymal transition has been indicated with induced expression of *Cdh1* while a loss in *Snai2* and *Tead4 l*evel in *Aplf* knockdown trophoblast stem cells undergoing differentiation have been observed (Varghese et al., 2021). An impaired differentiation of tropbhoblast stem cells due to the loss of APLF might contribute to the defect in implantation of mouse embryos.

Upon fertilization, the changes in histone composition and DNA methylation drive the reprogramming of the genome for further development. The canonical histone H3 is undetectable in mouse pre-implantation embryos. Erasure of oocyte-specific H3.3 mediated gene expression has been observed in mice embryo after fertilization (Akiyama et al., 2011). Global incorporation of H3.2 takes place within the transcriptionally silent heterochromatin regions immediately after fertilization. H3.1 and H3.3 accumulate at unusual heterochromatin and euchromatin regions. But, after 2-cell stage, H3.1 and H3.3 occupy their usual position within heterochromatin and euchromatin respectively (Akiyama et al., 2011). The expression of H3.3A, H3.2, and H3.1 are undetectable whereas H3.3B, Centromere Protein A (CENPA), and the H3-H4 chaperone CAF1 express throughout pre-implantation development, with relatively high expression in zygote soon after the fertilization. During pre-implantation embryogenesis, expression of canonical H2A appeared to be predominantly high in the hatched blastocyst, the variants like H2AX, H2AZ, H2AJ, H2AY1, and canonical H2B are expressed relatively more in mouse blastocyst (Kafer et al., 2010). Thus, histone composition contributes to the compaction or decompaction of chromatin that regulate the change in gene expression pattern associated with the development of embryo after fertilization.

The development and hatching of the embryo allow it to adhere to the walls of the uterine horn within the mother’s womb and thereby initiates the post-implantation development of the embryo.

### Gastrulation

Gastrulation is the stage where definitive primary germ layers-endoderm, mesoderm, and ectoderm are specified from a single layer of the epiblast present in the blastocyst stage of mammalian embryonic development. This stage is critical as the cells exit from a naïve state of pluripotency, become primed and poised towards germ layer specification (Lawson et al., 1991). Post-blastocyst stage, the inner cell mass (ICM) within the blastocyst gives rise to epiblast and primitive endoderm. The primitive endoderm differentiates to visceral endoderm (VE) and parietal endoderm (PE) whereas the epiblast generates ectoderm (from anterior epiblast) and primitive streak (from posterior epiblast). The primitive streak further differentiates into mesoderm and endoderm. Each of the primary germ layers specifies different tissue lineages. Embryonic endoderm differentiates to epithelial linings of the gut and respiratory tract whereas embryonic ectoderm gives rise to epidermis, Central Nervous System (CNS), eyes, internal ears. The embryonic mesoderm differentiates into connective tissues and constitutes a major part of internal organs. The event of gastrulation encompasses a number of evolutionarily conserved cellular movements viz internalization, epiboly, convergence, and extension (Keller, 2005). These morphogenetic cellular movements are achieved by changes in cell shape, directed migration, and cell divisions. Nodal/TGF*β*, BMP, FGF and WNT/*β*-catenin signaling pathways are involved in entailing specific germ layer fates (Morgani and Hadjantonakis, 2020). The chromatin accessibility determines the lineage specific gene activation and repression. Morphogen and signaling induced transcriptional regulation is facilitated by the open accessible chromatin. However, very little is known about chromatin accessibility mediated transcriptional control during early development. Histone variants and histone chaperones could possibly be facilitating the chromatin accessibility during gastrulation.

The optimal concentration, turnover and ratio of histones affect both the process and the patterning of the embryo during gastrulation. Mouse embryonic stem cells (ESCs) derived from the inner cell mass of blastocyst have reduced histone H3 content than that of cells differentiated from them (Karnavas et al., 2014). Expression analysis in mouse embryos shows an increase in H3.3 expression at gastrulation concomitant with its deposition by HIRA for the progression of mesodermal differentiation. HIRA is expressed within the extraembryonic-embryonic boundary and in the proximal ectoderm. Targeted mutation of *Hira* leads to mesodermal defects in mouse gastrulation embryos (E6.5–E7.5) by the loss of paraxial mesendodermal structures such as notochord and somite. Consequently, the neuroepithelial patterning alters and disorganizes before embryonic lethality by E10-E11 (Roberts et al., 2002). Mutant embryos exhibited distorted primitive streak marking their initial requirement in gastrulation (Roberts et al., 2002). The replicative counterpart of histone H3, H3.2, and its chaperone CAF1 is essential for cell survival, patterning, and segment identity in *Drosophila*. CAF1 mediated polycomb silencing determines the homeotic transformation by regulating *Senseless* genes. Mutations in CAF1-RBAP46/RBAP48 lead to suppression of *Senseless* overexpression, which is essential for eye and wing development in the fly. In *X. laevis*, inhibition of replicative H3.2 incorporation by dominant-negative CAF1-P150 or H3.2 morpholino results in arrest at mid blastula transition or early gastrulation, respectively, (Quivy et al., 2001; Szenker et al., 2012).

In mice, the histone H2A-H2B chaperone NAP1 have a critical role in embryo patterning and in determining the position of primitive streak by regulating the polarization and migration of cells in anterior visceral endoderm (AVE), a group of extra-embryonic cells (Rakeman and Anderson, 2006). NAP1 regulates WASP-family verprolin homologous protein (WAVE)- mediated actin branching in axis specification and morphogenesis in the mouse embryo. Furthermore, NAP1 regulated WAVE complex (WRC) are involved in migration of mesoderm and endoderm and neural tube closure. Several cellular movements are affected in the NAP1 mutant, which includes migration of AVE, definitive endoderm, and mesoderm. However, the movement is not completely blocked in any of these cases (Rakeman et al., 2006). In agreement with this, the expression of H2A variant, H2A.Z in *Xenopus* embryo found to reach its peak at gastrulation, unlike early mouse development where H2A.Z transcribe from the 2-cell stage. It has been reported that deposition of H2A.Z is important for proper mesoderm formation and its depletion leads to blastopore closure failure and results in embryo with shortened trunks and deformed neural plates at the tail bud stage (Ridgway et al., 2004). The *H2a.z* null mice die at around gastrulation (Faast et al., 2001). In *Zebrafish*, transient depletion of *H2afv* leads to DNA hypermethylation during embryonic patterning in particular during somitogenesis (Madakashira et al., 2017).

Different signaling mechanisms aided by an intricate network of transcription factors and epigenetic modifications dictate the differentiation of the embryo into distinct tissue specifications. So next, we focus on how histone chaperones execute their impact on this fate specification and functioning.

### Differentiation Events During Organogenesis

#### Neural Differentiation

After gastrulation, the embryo undergoes an event called neurulation that involves the development of the nervous system. During neurulation, the ectoderm differentiates into dorsal ectoderm, that becomes specialized to cells present on the outer surface of the body, and neural ectoderm which gives rise to the nervous system of the embryo. Adult stem cells present in the nervous system, called neural stem cells, proliferate and give rise to neurons and glia through a well-regulated process called neurogenesis. The proliferation and differentiation of neurons from the Neural Progenitor Cells (NPCs), specification of the subtypes, migration of immature neurons to their respective destination, maturation, and establishment of neuronal connectivity are precisely regulated key events in neural development.

Several studies have demonstrated the role of histone H3, H3 variants, and chaperones in neurogenesis. H3.3 incorporation maintains the level of H3K27me3 at the promoters of genes that regulate the development of neural tube mesenchyme and ear in mice (Banaszynski et al., 2013). Interestingly, histone chaperone ASF1, HIRA, and DAXX associated with H3.3 incorporation have been implicated in diverse event of neural development. In post-mitotic neurons, the replication-independent deposition of the histone H3.3 variant by the HIRA complex is a key nucleosome replacement mechanism regulating gene transcription. HIRA regulates the *ß*-catenin pathway, which plays an important role in regulating the developmental program and can direct progenitors to proliferate or differentiate (Zechner et al., 2003). HIRA expressed in embryonic cerebral cortex and NPCs, recruits H3K4 trimethyltransferase, SET Domain protein 1A (SETD1A), at the *ß*-catenin promoter to increase H3K4me3 level resulting in increased *ß*-catenin expression. Hence, perturbation of *Hira* leads to restricted proliferation of NPCs, increase in terminal mitosis and cell cycle exit and ultimately leads to premature neuronal differentiation (Li and Jiao, 2017). Depletion of another H3.3 chaperone, ATRX in NPCs causes replicative stress and DNA damage at telomeres and pericentric heterochromatin in mitotically active neurons (Watson et al., 2013). DAXX appeared to be crucial for corticogenesis as it associates with Calcium-dependent dephosphorylation mediated H3.3 loading and transcription upon neuronal activation (Michod et al., 2012).

In mammals, *Nap1* encodes for three neuron-specific genes *Nap1l2*, *Nap1l3,* and *Nap1l5,* whereas other transcript variants are ubiquitous. Among them NAP1L1 has been implicated in mouse brain and neocortical development in particular (Qiao et al., 2018). In mice, NAP1L1, is expressed in the developing cerebral cortex and NPCs. However, upon differentiation, the expression progressively declines in neural progenitors. *Nap1l1* knockdown mice exhibit premature neuronal differentiation and decrease in progenitor pool suggesting its role in corticogenesis from E13.5 to E16.5. In addition, NAP1L1 also promotes radial glial cell proliferation. Not only NAP1L1 modulates neuronal differentiation but also the cell cycle exit in neuronal progenitors. Mechanistically, NAP1L1 regulates the Ras Association Domain Family Member 10 (RASSF10) expression by promoting SETD1A mediated H3K4 methylation on the *Rassf10* promoter, hence controls NPCs differentiation (Qiao et al., 2018). In *Drosophila*, depletion of neuron-specific NAP1, NAP1L2, leads to embryonic lethality from the mid-gestation phase exhibiting ectoderm defects, and the neural tube defects are attributed by enhanced proliferation of neural precursors (Rogner et al., 2000; Lankenau et al., 2003).

Similar to NAPLl1, depletion of NAP1L2 in mice not only affects the differentiation but also enhances the proliferation and apoptosis of the cells. The binding of NAP1L2 with H3 and H4 in the nucleus of neuronal cells increases the H3 acetylation (H3K9/14) at the Cyclin dependent kinase inhibitor 1C (*Cdkn1c*) promoter. The expression of CDKN1C increases during neuronal differentiation that correlates with the specific recruitment of NAP1L2 for histone acetylation (Attia et al., 2007).

Recently a study has investigated the functional implication of H2A histone variant H2A.Z in embryonic neurogenesis (Shenet al., 2018). Interestingly, *H2a.z* knockdown phenotype in mice appeared to be the same observed for H2A-H2B chaperone NAP1 transcript variants. Depletion of H2A.Z promotes proliferation of neural progenitors and inhibits neuronal differentiation. However, the *H2a.z* knockout mouse exhibits abnormal dendrites with cortical development. H2A.Z recruits methyl-transferase, SETD2 to enhance H3K36me3 levels at NK2 Homeobox 4 (*Nkx24*) promoter hence increasing its expression, which ultimately regulates cortical neuron expansion (Shenet al., 2018). Moreover, in mice, the core histone chaperone binding protein Acidic Leucine rich Nuclear Phosphoprotein 32 (ANP32) family, comprising of ANP32A, ANP32B, and ANP32E, is specifically involved in supply and eviction of H2A.Z and have a subtle role in embryogenesis. *Anp32b* ^-/-^ mice exhibit mild and sporadic craniofacial abnormalities, possess expanded ventricle and inner ear cavities, and palate closure defects suggesting its implication in brain development during embryogenesis (Reilly et al., 2011). Similarly, depletion of H2A.Z.1 subdues gliogenesis and results in reduced astrocyte differentiation in mice. It also interacts with histone chaperone ASF1A to regulate H3K56ac, which in turn modulates the expression of Folate Receptor Alpha 1 (FOLR1), a signal-transducing component of the JAK-STAT pathway, thereby regulating gliogenesis (Su et al., 2018).

#### Hematopoietic Differentiation

Embryonic hematopoiesis occurs in two distinct waves - primitive and definitive hematopoiesis which takes place in the yolk sac and dorsal aorta or equivalent structure respectively. The primitive wave produces primitive erythrocytes for oxygen transport and hence critically important for the survival of the embryo. During the first wave, in the yolk sac, the myeloid cells are also produced, which migrates to CNS and skin to differentiate to microglia and Langerhans cells. On the other hand, definitive wave generates multipotent hematopoietic progenitors from mesoderm derivatives. These cells are produced once in a lifetime. Different lineages of blood cells are produced continuously from the pool of hematopoietic stem cells (HSCs). HSC expansion and differentiation occurs in fetal liver, subsequently the progenitor exit and migrates to spleen for further differentiation into myeloid and lymphoid subset. The progenitors develop from hemogenic endothelium (HE), which undergoes endothelial to hematopoietic transition to give rise to HSCs.

HIRA regulates angiogenesis in endothelial cells by H3.3 mediated K56 acetylation on genes implicated in angiogenesis (Dutta et al., 2010). Interaction of HIRA with the chromatin remodeler Brahma-Related Gene 1 (BRG1) regulates the transcription factor Runt-related Transcription Factor 1 (RUNX1) by the incorporation of H3.3 in the enhancer element of *Runx1* and regulates the downstream targets Spi-1 Proto-Oncogene/Hematopoietic Transcription Factor (*Pu.1*), Growth Factor Independent 1 Transcriptional Repressor (Gfi1), Gfi2 and GATA Binding Protein 2 (*Gata2*) to generate hematopoietic stem cells from hemogenic endothelium differentiated from mouse ESCs (Majumder et al., 2015). Furthermore, the erythroid lineage differentiation factors including Erythroid Kruppel Like Factor (EKLF), GATA1, and *ß*-globin are regulated by HIRA. EKLF recruits HIRA to the *β-globin* locus for the incorporation of H3.3 to retain the open chromatin state by forming HIRA/ASF1A/H3.3/EKLF complex (Soni et al., 2014). Recent study has shown that HIRA can dictate differentiation of Chronic Myeloid Leukemia cells to megakaryocytes via MKL1/GATA2/H3.3 axis (Majumder et al., 2019). Conditional knockout of *Hira* in hematopoietic cells exhibits a dramatic reduction in HSCs in the bone marrow resulting in thrombocytopenia, lymphocytopenia, and anemia. Massive depletion of T and B-lymphocytes, monocytes and platelets, and RBCs is observed in *Hira* knockout mice. However, the fetal hematopoiesis is normal, although they demonstrate impaired reconstitution capacity due to defective chromatin accessibility (Chen et al., 2020). Additionally, HIRA has been implicated in heart development by regulating the troponins (Troponin I2, TNNI2, and Troponin T3, TNNT3) involved in cardiac contractility and EPH Receptor A3 (EPHA3), a vital gene for the fusion of muscular ventricular septum and the atrioventricular cushions. Conditional knockdown of HIRA in cardiogenic mesoderm in mice leads to defects in ventricular and atrial septation, surface edema, and embryonic lethality (Dilg et al., 2016).

Interestingly, a recent study has demonstrated the requirement of NAP1L3 in the maintenance and differentiation of HSC. NAP1L3 is highly expressed in mouse hematopoietic stem cells concerning the downstream progenitors proposing its potential function in primitive hematopoiesis. The perturbation of these genes impairs colony-forming capacity both *in vitro* and *in vivo* after transplantation. In agreement with this, downregulation of human NAP1L3 in umbilical cord blood derived HSCs induces G0 arrest of cell cycle and affects the expression of E2 transcription factor (E2F) and MYC targets as well as Homeobox A cluster 3 (HOXA3) and Homeobox A cluster 5 (HOXA5) (Heshmati et al., 2018). Zygotic depletion of NAP1L in *Xenopus*, affects *α*-globin expression in ventral blood island and result in reduction in transcript level of precursor genes Erythroid differentiation factor (SCL) and XAM1 (ortholog of RUNX1) (Abu-Daya et al., 2005).

Other histone chaperones including the P60 subunit of CAF1 (CHAF1B), which assembles histones H3.1/H4 heterodimers at the replication fork during the S phase and DEK, a nuclear phosphoprotein have been implicated in the maintenance of adult hematopoiesis. DEK expression is enhanced by inflammatory stimuli and it also increases the self-renewal and repopulating capacity of hematopoietic stem cells and subdues the number of hematopoietic progenitor cells. Perturbation of these chaperones results in hematological malignancies in adults (Capitano and Broxmeyer, 2017; Volk et al., 2018).

#### Muscular Differentiation

Skeletal muscles are derived from myogenic progenitor cells (MPCs) and are regulated by a family of highly related transcription factors together known as the myogenic regulatory factors (MRFs) (Berkes and Tapscott, 2005). It includes Myogenic Factor 5 (MYF5), MyoD, myogenin, and MYF6 (also known as MRF4). They function along with the MEF2 family of MADS-box transcription factors, which lacks any myogenic activity alone (Molkentin et al., 1995; Wang et al., 2001; Blais et al., 2005). During muscle differentiation, proliferating myoblasts exit the cell cycle concomitant with muscle-specific gene activation to differentiate to multinucleated myotubes (Sartorelli and Caretti, 2005).

The transcription factors, MYOD and Myocyte Enhancer Factor 2 (MEF2) recruit ATP-dependent chromatin remodellers and histone modifying enzymes to activate the muscle-specific genes. In proliferating myoblasts *in vitro*, HIRA enhances the transcriptional activity of MEF2, which is opposed by the Calcineurin Binding Protein 1 (CABIN1), which is a component of the HIRA-containing complex. CABIN1 via its N-terminal domain interacts with C-terminus of HIRA and result in the suppression of MEF2/HIRA mediated transcription. The repression of MEF2/HIRA is removed by the reduction in the level of CABIN1 upon differentiation of myoblasts. The association of ASF1 with HIRA enhances the MEF2 transcriptional activity thereby regulating other muscle-specific genes like myogenin (Yang et al., 2011).

The myogenic activation of MyoD is mediated by replication independent histone deposition. The incorporation of non-replicative H3.3 on the promoter and regulatory elements of MyoD by HIRA and ASF1B enables chromatin to become more permissive for RNA polymerase II to access for the transcriptional activation (Yang et al., 2011). The same group has investigated that the phosphorylation of serine (S) 650 in human HIRA mediated by AKT Serine/Threonine Kinase 1 (AKT1) is key in regulating muscle gene transcription during cellular differentiation (Yang et al., 2016). Transcriptional regulation of *MyoD* is also mediated by the coordinated interplay of histone chaperone-PolII-demethylase complex. It has been reported that histone chaperone SPT6 in association with PolII and H3K27 demethylase KMD6A, counteracts the repressive mark H3K27me3 on Myod for its activation. The depletion of SPT6 in Zebrafish embryos fails to undergo myogenic differentiation due to the enrichment of H3K27me3 on the Myod gene (Wang et al., 2013).

Histone chaperone can associate with long noncoding RNA (lncRNA) in regulating constitutive heterochromatin establishment during myogenesis where chromocenters form clusters progressively, which lead to heterochromatin compartment establishment (Park et al., 2018). The muscle-specific lncRNA, ChRO1 associate with DAXX/H3.3 and bind to ATRX, forms a stable complex at chromocenters for the elevation of satellite RNA and subsequently the fusion of chromocenters and establishment of heterochromatin compartment (Park et al., 2018).

Our insights on embryonic development are often from studies performed *in vitro* in the ESCs. The source for an embryo proper to develop into an entire organism lies in a distinct set of cells called the inner cell mass, which is present during the blastocyst stage of the pre-implantation development. The pluripotent nature of those cells has been widely exploited *in vitro* by the generation of ESCs. To avoid ethical concerns, scientists have generated pluripotent stem cells similar to ESCs independent of an embryo. So, it would be important to have a look at how histone chaperones could regulate this character of cells, which forms the basis of the development of an entire embryo.

### Pluripotency and Reprogramming

Pluripotent cells have a unique potential to self renew and to differentiate into all three cell lineages. Hyperdynamicity in the association of chromatin proteins by controlled epigenetic modifications is a hallmark of pluripotency (Meshorer et al., 2006)., The pluripotent cells demonstrate an open chromatin structure similar to the zygote, which endows its ability to give rise to all cell types *in vitro* and *in vivo*. With the advent of *generation* of induced pluripotent stem cells (iPSC) with the ectopic expression of transcription factors–Octamer binding transcription factor 4 (OCT4), SRY-Box transcription factor 2 (SOX2), Kruppel like factor 4 (KLF4), and Proto-oncogene c-MYC, understanding of switching the cell fate from terminally differentiated cells to pluripotency by altering the chromatin state became more refined (Takahashi and Yamanaka, 2006). Even after more than a decade to its discovery, the efficiency or time required to derive these cells is highly inefficient. This is possibly because of the epigenetic barrier that combats change in cell fate. Basically, generation of iPSC entail a global epigenetic remodeling from one state to another. In the context of somatic cell nuclear transfer (SCNT) and iPSCs generation, epigenetically regulated chromatin accessibility becomes the barrier in nuclear reprogramming to preserve the cell identity.

Pluripotent ESCs are isolated from the inner cell mass of the blastocyst and cultured in presence of exogenous factors to maintain the undifferentiated state. The most common factor to culture mouse ESC is the addition of Leukemia Inhibitory Factor (LIF) while it is basic fibroblast growth factor (FGF2) that could retain the undifferentiated state of human ESCs. The removal of these growth factors lead to spontaneous differentiation of ESCs while customized cocktails of growth factor and other signaling molecule could drive their differentiation to specific lineages. Studies on maintenance of the ESCs in undifferentiated vs. differentiated states have been conducted and multiple histone chaperones have been implicated in the context of pluripotency of ESCs. Both replicative and non-replicative histone H3 and its chaperones are indispensable for self-renewal and maintenance of pluripotency in ESCs.The non-replicative H3.3 chaperone HIRA is predominantly expressed in the promoters of genes in mouse ESC. The deposition of H3.3 is critical upon differentiation for controlling the expression of bivalent genes whereas it is dispensable in mouse ESC for its self-renewal (Goldberg et al., 2010; Banaszynski et al., 2013). HIRA–mediated H3.3 deposition enriches the H3K27me3 mark at the bivalent gene promoters for PRC2 occupancy and then its depletion leads to the upregulation of genes involved in trophectoderm lineage specification in mouse ESC (Banaszynski et al., 2013). HIRA depleted mouse ESCs undergo accelerated differentiation due to the presence of increased amount of unbound histones (Meshorer et al., 2006). HIRA acts along with prohibitin for the maintenance of pluripotency of human ESCs by enhancing the transcription of the isocitrate dehydrogenase gene (Zhu et al., 2017). ASF1A regulates the expression of pluripotency related genes including OCT3/4, NANOG, SOX2, and DNMT3B in human ESC. ASF1A is involved in H3K56 acetylation, a histone mark prevalent in hESC, indicating the possibility of its involvement in the regulation of transcription network of pluriportency-related genes to maintain the undifferentiated state of pluripotency (Gonzalez-Muñoz et al., 2014). ASF1A through the interaction with histone when recruited along with transcription factors to the bivalent promoters, signature of ESCs, could induce the lineage specific gene transcription in mouse ESCs (Gao et al., 2018). The H2A-H2B chaperone NPM3 is highly expressed in the pluripotent cells and is involved in mouse ESC proliferation and maintenance (Motoi et al., 2008).

CAF1 is essential for heterochromatin formation in ESCs. During differentiation of mouse ESCs, the replicative chaperone CAF1, in association with proliferating cell nuclear antigen (PCNA) facilitates the formation of facultative heterochromatin by silencing the pluripotency genes, Oct4, Sox2, and Nanog as well as by establishing H3K27me3 mark at these gene promoters (Cheng et al., 2019). This is in support of acquisition of pluripotency and maintenance of Heterochromatin protein 1 gamma (HP1 γ) in association with CAF1 (Zaidan et al., 2018) and involvement of CAF1 in heterochromatin organization in pluripotent ESCs (Houlard et al., 2006). Depletion of histone chaperone SPT6 reduces the expression of pluripotency factors, increased transcription of cell lineage affiliated regulators and morphological changes inductive of early cell differentiation. SPT6 interacts with PRC2 core subunit SUZ12 and counteracts repression by H3K27trimethylation recruitment at super enhancers in ESCs (Wang et al., 2017; Robert, 2017). The H2A-H2B chaperone NAP1L1 recruited by Forkhead box A2 (FOXA2), coupled with chromatin remodeler SWI/SNF and INO80 and H2A.Z mediate nucleosome depletion during ESC differentiation (Li et al., 2012). Based on the conditional OCT4 depletion, two isoforms of H1 specific SET chaperones in ESC have been found, SET*α*/*β* switch, which is important for ESC differentiation. SETα is responsible for the maintenance and proliferation whereas SET *ß* in the differentiation of ESCs (Edupuganti et al., 2017).

**FIGURE 3:**
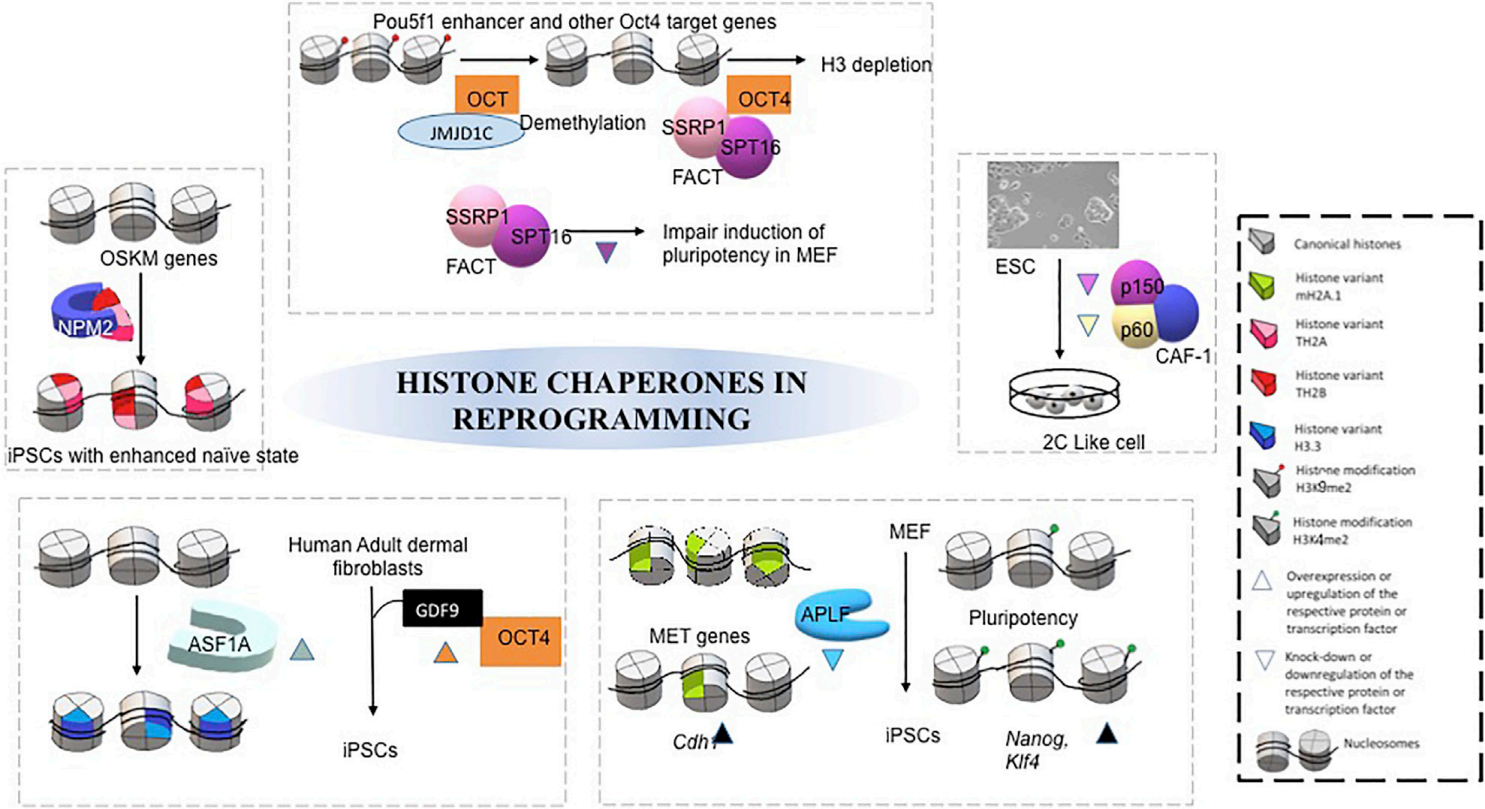
Mechanism of different histone chaperones in facilitating acquisition of pluripotency by reprogramming. Each separate box represents mechanism by which different histone chaperone facilitate reprogramming, mediated by different transcription factors, histone modifications, growth factors and histone variants (Gonzalez-Muñoz et al., 2014; Ishiuchi et al., 2015; Shakya et al., 2015; Fernández-Rivero et al., 2016; Syed et al., 2016; Shen Z et al., 2018).

Seminal studies have implicated various histone chaperones in the acquisition of pluripotency and reprogramming of somatic cells and ESCs (Figure 3). Deregulation of histone chaperones either its suppression or activation directly or indirectly regulates chromatin template processes in facilitating reprogramming and pluripotency. Ishiuchi T and others induced the generation of totipotent 2C like cells by downregulation of either of P150 or P60 subunits of CAF1 in ESCs (Ishiuchiet al., 2015). *In vitro* generated induced totipotent 2C like cells exhibit a similar transcriptional profile as 2-cell stage embryo having a higher degree of reprogramming capacity than the formation of ESC-like cells upon nuclear transfer (Ishiuchiet al., 2015). Following this study, the same group has demonstrated CAF1-P150 and CAF1-60 as a major barrier to iPSC derivation. The suppression of either of these subunits enhances the efficiency of reprogramming, which has been demonstrated by inducing the iPSC generation by downregulation of CAF1-P150 and CAF1-60 in mouse embryonic fibroblasts (MEFs) (Cheloufi and Hochedlinger, 2017).Additionally, the core histone chaperone APLF has been found to have a role in the induction of pluripotency. Downregulation of APLF accelerates the reprogramming process and induces the efficiency in the generation of pluripotency in MEFs. The loss of APLF leads to the loss of repressive mark MacroH2A.1 from the promoter of mesenchymal-to-epithelial transition (MET) specific gene, E-cadherin (Cdh1), and the incorporation of H3K4me2 mark at the promoters of pluripotency genes Nanog and Klf4, enhanced the efficiency of iPSC generation (Syed et al., 2016). The overexpression of a non-replicative H3.3 chaperone, ASF1A, and OCT4 in human adult dermal fibroblast (hADFs) supplemented with oocyte-specific growth factor GDF9 reprogram the somatic fibroblast to pluripotent cells (Gonzalez-Muñoz et al., 2014). Recently, the chaperone complex Facilitate Chromatin transcription (FACT) has been implicated in inducing pluripotency by facilitating nucleosome disassembly that provides access to chromatin for the transcription factors during the transcription. FACT-mediated transcriptional activation is required for an early stage reprogramming and for the recovery from stress associated with passaging of mouse ESCs whereas it is dispensable for the maintenance and proliferation of these cells (Shen T et al., 2018). FACT interacts with OCT4 by having an association with Jumonji Domain Containing 1C (JMJD1C) and concomitant loss of H3K9me2, which leads to subsequent depletion of local H3 (Shakya et al., 2015). Hence, FACT is involved with OCT4 to mediate nucleosome eviction. Moreover, depletion of SPT16, one among the subunits of FACT, impairs the induction of pluripotency in MEFs. However, the proliferation was not affected. The H2A-H2B histone chaperone NPM2 is associated with TH2A and TH2B by modulating OSKM transcription factors, in generating iPSC with enhanced naïve character. Phosphorylation of NPM2 establishes the open state chromatin and enhances the reprogramming capacity of the cells (Fernández-Rivero et al., 2016). A detailed review has been published on the role of chaperones in pluripotency (Fernandes et al., 2019). A plethora of studies summarized in this section has given mechanistic insight into the function of histone chaperones in pluripotency (Figure 3), which could contribute to the generation of patient-specific iPSCs and disease models.

**FIGURE 4:**
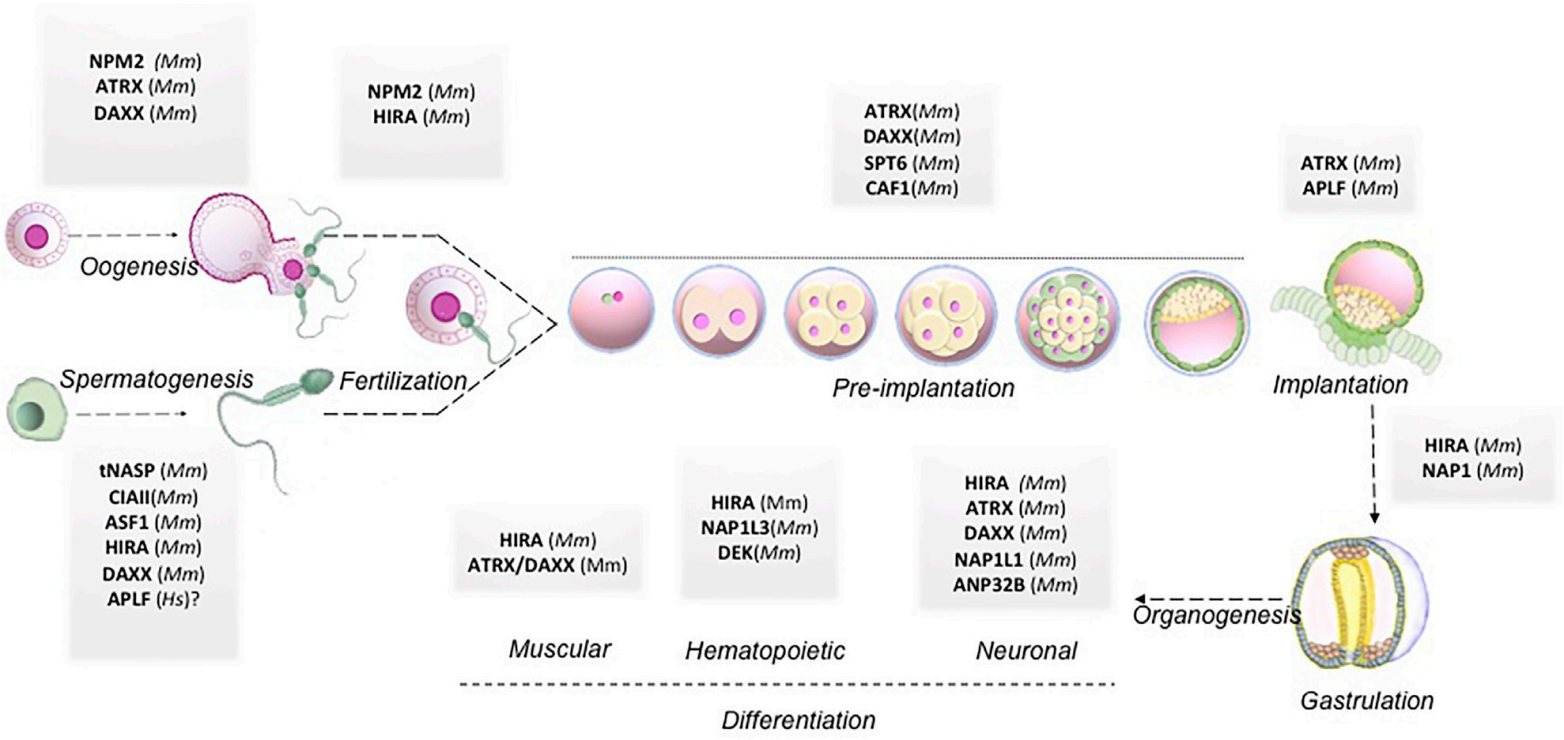
Histone chaperones in mammalian embryo development. Schematic representation of functional implication of different histone chaperones involved in different stages of embryo development right from gametogenesis to organogenesis. Abbreviations: Mm, *Mus musculus*; Hs, *Homo sapiens.*

The review elaborated on how histone chaperones associated with respective canonical or non-canonical histones could regulate different stages of pre-and post-implantation development. The same along with the respective histone chaperones knock out phenotypes have been summarized in Table 1. A pictorial representation has been included to demonstrate the histone chaperones implicated at different stages of mammalian development mentioned hereby (Figure 4).

**TABLE 1:** Histone chaperones: Types of histones, role in development and KO phenotypes.

Histone chaperones	Preferred histones	KO phenotype	Key role in development
Chromatin Assembly Factor 1 (CAF1)	H3.1/H4	Early embryonic lethality at the stage of compaction (16 cell stage) in *M.musculus* (Houlard M et al., 2006)	• Involves in spermatid maturation CAF1 P180 and CAF1 P75 involves replacement of histones by protamines in sperm chromatin in *D. melanogaster* (Doyen et al., 2013; Doyen et al., 2015)
			• CAF1 P180 subunit is required for GSC maintenance of female germline in *D. melanogaster* (Clémot et al., 2018)
			• Protects hypomethylated preimplantation mouse embryos from retrotransposons (Hatanaka Y et al., 2015)
			• CAF1 P150 is essential for gastrulation in *X. laevis* (Quivy et al., 2001; Szenker et al., 2012)
	H3.2/H4	CAF1 P180 null are hemizygous lethal in *D. Melanogaster* (Song et al., 2007)	• Required for establishment of bilateral asymmetry and cell division in *C.elegans* nervous system (Nakano et al., 2011)
			• CAF1 in association with PCNA facilitates formation of facultative heterochromatin by silencing the pluripotency genes (Cheng et al., 2019)
			• Involves in heterochromatin formation in pluripotency acquisition and maintenance in ESC (Houlard et al., 2006; Zaidan et al., 2018)
			• Depletion of either of CAF1 P150 or CAF1 P60 enhances reprogramming capacity and iPSC generation from murine fibroblast (Cheloufi et al., 2015) and generation of totipotent 2C-like cells *in vitro* (Ishiuchi et al., 2015)
Histone Cell Cycle Regulator (HIRA)	H3.3/H4	Early embryonic lethality at E10 in *M. Musculus* (Roberts et al., 2002)	• Decondensation of sperm chromatin during male pronucleus formation at fertilization in *D. melanogaster* (Bonnefoy et al., 2007) and in *M.musculus* (van der Heijden et al., 2005)
			• HIRA mediated H3.3 incorporation in gastrulation and mesoderm formation in *X. laevis* (Szenker et al., 2012)
			• Involves in regulation of cortical neurogenesis by modulating the expression of beta-catenin (Li and Jiao, 2017)
			• Critical regulator of hematopoiesis and heart development (Dilg et al., 2016; Chen et al., 2020)
			• Involves in trophectoderm lineage specification in mESC *in vitro* (Banaszynski et al., 2013) and in maintenance of pluripotency in hESC (Zhu et al., 2017)
Anti Silencing Factor (ASF1)	H3.1/H4	*Asf1a* null embryos dies at mid-gestation (E9.5) in *M. Musculus* (Hartford et al., 2011)	• ASF1 assists HIRA in the deposition of H3.3 in sperm chromatin for the male pronucleus formation during fertilization (Horard et al., 2018)
			• ASF1B regulates the timing of meiotic entry in spermatocytes during spermatogenesis (Messiaen et al., 2016)
	H3.2/H4		• ASF1A in association with HIRA mediates the incorporation of H3.3 for MYOD expression (Yang et al., 2011)
	H3.3/H4	However, *Asf1b* null female is subfertile and affects the timing of meiotic entry and gonad development (Messiaen et al., 2016)	• ASF1A involves in reprogramming of adult dermal fibroblasts to iPSC (Gonzalez-Muñoz et al., 2014)
			• ASF1A regulate the nucleosome disassembly at the bivalent promoters in mESCs to induce lineage specific differentiation (Gao et al., 2018)
			• UNC85 involves in postembryonic neuroblast development in *C. elegans* (Grigsby and Finger, 2008)
Death Domain Associated Protein (DAXX)	H3.3/H4	Early embryonic lethality at E9.5 in *M. Musculus* (Michaelson et al., 1999)	• Regulates apoptosis in early embryo (Michaelson et al., 1999)
	CenH3		• Involved in corticogenesis (Michod et al., 2012)
			• Establishment of heterochromatin in association with ATRX and lncRNA during myogenesis (Park et al., 2018)
ATRX	H3.3/H4	Embryonic lethality at or around E9.5 in *M.musculus* (Garrick et al., 2006)	• Required for extraembryonic trophoblast formation in *M. musculus* (Garrick et al., 2006)
			• Maternally derived ATRX is important for chromosomal segregation and genomic stability in oocyte (Baumann et al., 2010)
			• Silencing repetitive elements in mouse pre-implantation embryo and mESC (He et al., 2015)
			• Establishment of heterochromatin in association with DAXX and lncRNA during myogenesis (Park et al., 2018)
			• Involves in DNA damage repair and heterochromatin formation at telomeres in mitotically active neurons (Watson et al., 2013)
Nuclear Autoantigenic Sperm Protein (NASP)	H1	Early embryonic lethality in *M. musculus* and null mice survive until the stage of blastocyst (Richardson et al., 2006)	• Regulation of cell cycle progression during spermatogenesis in *M. musculus* (Alekseev et al., 2005)
	H3/H4		• Deposition of sperm chromatin components onto the fly sperm genome during packaging of male genome (Doyen et al., 2015)
Nucleosome Assembly Protein 1 (NAP1)	H2A/H2B	Either embryonic lethal or less viable in *D. melanogaster* (Lankenau et al., 2003)	• NAP1 is critical for embryo patterning and in determining the position of primitive streak in *M. musculus* (Rakeman and Anderson, 2006)
		*Nap1l1* KO causes abnormal embryonic neurogenesis in *M. Musculus* (Qiao et al., 2018)	• NAP1L1 is required for corticogenesis from E13.5 to E16.5 in mice (Qiao et al., 2018)
			• NAP1L3 is essential for the maintenance and differentiation of HSC in mice (Heshmati et al., 2018)
			• NAP1L1 is essential for ESC differentiation (Li et al., 2012)
Nucleoplasmin/Nucleophosmin (NPM)	H2A/H2B	*Npm2a* and*Npm2b* mutant exhibits early embryonic lethality in *D.rerio* (Cheung et al., 2018)	• Involves in decondesation of sperm chromatin after fertilization in *X. laevis* (Platonova et al., 2011) and mice (Inoue et al., 2011)
		*Npm2* KO females exhibits fertility defects due to failed pre-implantation development in *M. Musculus* (Burns et al., 2003)	• NPM1 is required for progenitor expansion during primitive hematopoiesis in *Zebrafish* (Barbieri et al., 2016)
			• NPM3 involves in mESC proliferation and maintenance (Motoi et al., 2008)
			• NPM2 establishes open chromatin state and induction of iPSC with more naïve nature (Fernández-Rivero et al., 2016)
SPT6 Homolog, Histone chaperone and Transcription elongation factor (SUPT6H)	H2A-H2B	Embryonic lethality during pre-implantation development in *M. Musculus* (Oqani al., 2019)	• Involves in myogenic differentiation by regulating MYOD in *Zebrafish* (Wang et al., 2013)
			• SPT6 in association with PRC2 involves in maintenance of pluripotency (Robert, 2017)
Acidic Leucine rich Nuclear Phosphoprotein 32 (ANP32)	Core histones	*Anp32e* and *Anp32a* KO mice are viable and fertile	• Brain development (Reilly et al., 2011)
	H2A.Z	*Anp32b-/-* mice exhibits partially penetrant, perinatal lethal and exhibits mild and sporadic craniofacial abnormalities (Reilly et al., 2011)	
Aprataxin PNK Like Factor (APLF)	Core histones	*Aplf* KO mice are viable (Rulten et al., 2011; Tong et al., 2016)	• Knockdown of APLF in mouse fails to implant and induce early hatching *in vivo* (Varghese et al., 2021)
	Macro H2A		• Male infertility (Cheung et al., 2019)
			• Knockdown of APLF enhances iPSC generation in murine fibroblast (Syed et al., 2016)

## Conclusion and Future Directions

**FIGURE 5:**
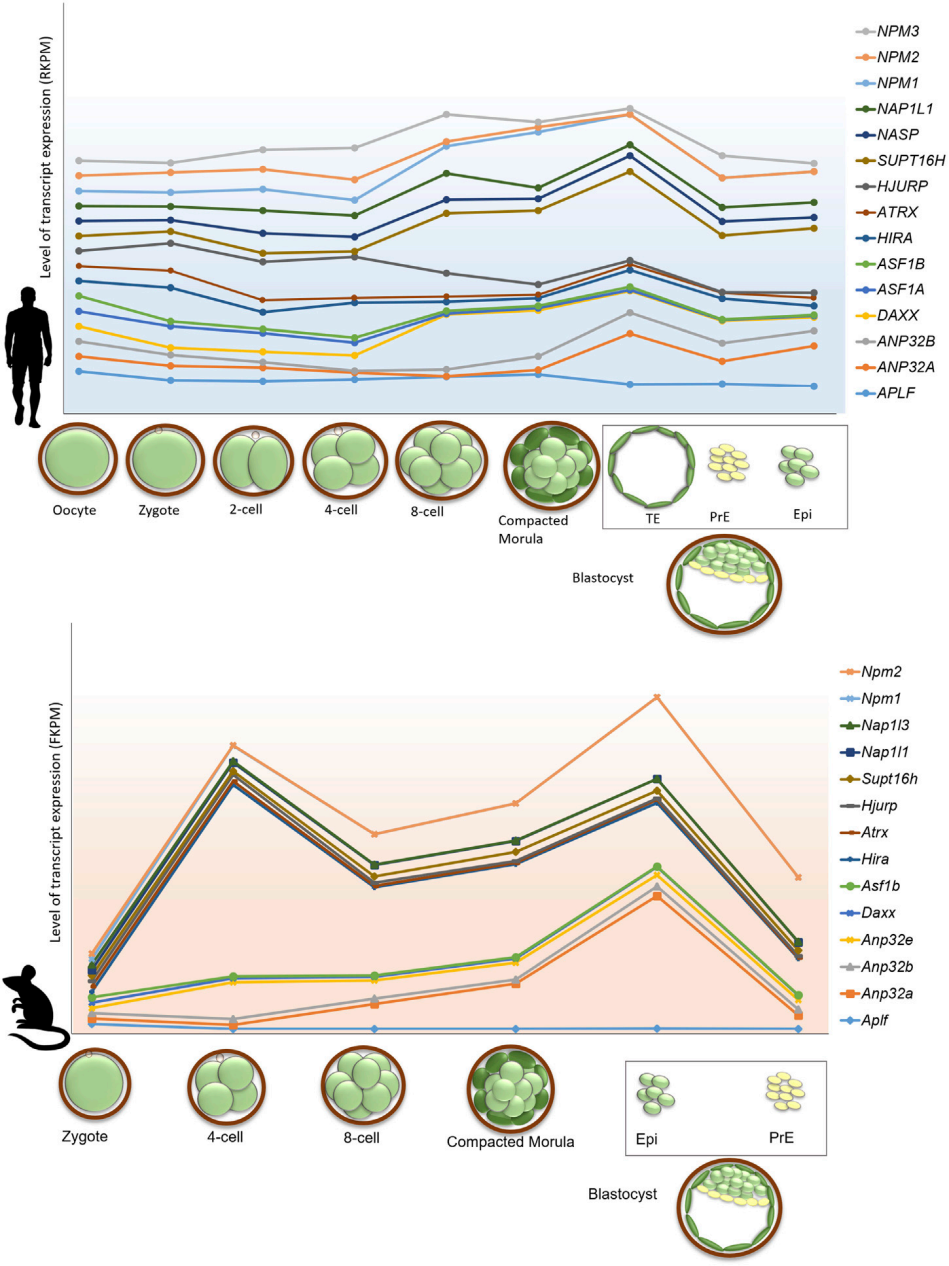
Gene expression profile of histone chaperones in pre-implantation stages in human and mouse embryo. Gene expression of histone chaperones between human and mouse exhibits difference in the expression pattern in different stages of pre-implantation embryos. Transcriptome profile of histone chaperones in pre-implantation stages of mouse (lower panel) and human (upper panel) embryos were extracted from and Boroviak et al., 2018 and Yan et al., 2013. Expression of histone chaperones in pre-implantation embryos were normalized with respect to oocyte and zygote in human and mouse, respectively. The *X*-axis represents the pre-implantation developmental stages; *Y*-axis represents the level of transcript expression of different chaperones. The level of transcript expression is represented in RPKM (Reads per kilobase of transcript per million reads mapped) inhuman and FPKM (Fragments per kilobase of transcript per million reads mapped) in mouse. The box represents cell types derived from blastocyst stage of the embryo. Abbreviations-TE, Trophectoderm; PrE, Primitive, Endoderm; Epi, Epiblast.

The regulation of gene expression during programmed switching of cell fate in developmental events relies on the dynamicity of chromatin and its accessibility for other chromatin proteins. In this review, we have discussed the involvement of histone chaperone-histone complexes in different stages of development right from gametogenesis to organogenesis. Although quite a significant portion remains to be deciphered, this concise inspection through the pages of histone chaperones in development indeed emphasized the proven presence of multi-faced characters of histone chaperones. Analysis of the single-cell transcriptomic data provided by Boroviak et al. and Yan et al. revealed the difference in the dynamicity of gene expression of histone chaperones in different pre-implantation stages in mice and humans (Yan et al., 2013; Boroviak et al., 2018). Furthermore, in mice, the gene expression of some of the histone chaperones follows the same expression pattern in different stages of pre-implantation embryos. For instance, the expression of *Atrx, Hira, Hjurp, Supt16h, Nap1l1, Nap1l3, Npm1, and Npm2* peaks at around 4-cell stage embryos and declines abruptly in the following stages and associate predominantly in the epiblast. On the contrary, no particular pattern of expression was observed for histone chaperones in human pre-implantation embryo stages (Figure 5). These findings indicate the relevance of the coordinated interplay of histone chaperones in embryogenesis. Given the differences in the chromatin affinity and interaction with its histone counterpart, histone chaperones can have a distinct function in a context-dependent manner.

Histone chaperones and ATP-dependent motor proteins, or to be precise, chromatin remodelers mediate chromatin assembly and disassembly. Throughout this review, we have tried to focus on how histone chaperones at different stages of development aid in the chromatin remodeling process by either incorporation or removal of active or repressive histone marks. The marks can be either in the form of canonical histones, histone variants or histone modifications. Studying development starting from gametogenesis to organogenesis is quite a herculean task due to absence of model system especially of human origin. Hence, discoveries or thorough studies are mostly prevalent in lower organisms baring mouse as the sole mammalian species that have significantly exploited. We have highlighted the differences in the function of these chaperones in species discussed in this review (Table 2). For these reasons, the detailed mechanism on how the histone chaperones are reorganizing the chromatin is yet to be physically accessed in different model systems. Our review mainly focuses on histone chaperones implicated in the development of mammals including mouse and human. However, the mechanistic studies have been extensively studied only in lower organisms. Meiosis, an integral phenomenon during gametogenesis, involves extensive chromatin remodeling. In yeasts, histone H2A-H2B and H3-H4 chaperones, NAP1 and Hir1, respectively can mediate exchange of individual histones within the nucleosome. Recruitment of these chaperones could activate the hotspots for meiotic recombination that help in its positioning (Storey et al., 2018). These chaperones are highly conserved in eukaryotes as well and hence might contribute in a similar fashion to the regulation of meiotic recombination machinery. ASF1 mediated H3K56ac is necessary for DNA replication in germline of *C. elegans* (Grigsby et al., 2009) while in mouse loss of one of its isoform ASF1B cause delay in initiation of meiosis resulting in defective gonad development both in male and female (Messiaen et al., 2016). Interestingly, alterations in the chromatin landscape associated during spermatogenesis have been investigated broadly that featured the role of histone chaperones. Sperm chromatin undergoes condensation and decondensation by loss or incorporation of protamines in exchange with histones. This mechanism is mediated by histone chaperones CAF1p75/NPM2/tNASP (Doyen et al., 2013; Rathke et al., 2014; Doyen et al., 2015). HIRA mediated incorporation of histone variant H3.3 promote the decondensation of chromatin contributing towards transcriptional activation of genes during oogenesis and spermatogenesis. So, depending on the incorporation, exchange or removal of histone marks by the histone chaperones, the chromatin landscape is modified either to a repressed or to an active state. In addition to this mechanistic interventions, histone chaperones like HIRA, APLF have been observed to be involved in other ways that aid in the transcriptional activation or repression of genes associated with different stages of development. During oogenesis, depletion of HIRA resulted in the change in *de novo* methylation status of the chromatin resulting in altered gene expression. APLF has been shown to influence the cellular transitions during pre-implantation development of mouse embryo thereby dictating the stages of further development. Also phosphorylation of histone chaperones like NPM2 or HIRA result in open chromatin that induce reprogramming and muscle gene transcription respectively. So, to summarize, the chromatin state responsive to the presence or absence of a histone chaperone, contribute to the transcriptional regulation of genes associated with different stages of development and is an outcome of multi-faceted functions of histone chaperones.

**TABLE 2:** Mechanistic regulation mediated by histone chaperones during development in multiple species.

Histone chaperones	Events in development	Mechanisms				
		Human	Mouse	*Xenopus*	*Drosophila*	*Zebrafish*
APLF	Spermatogenesis	Affects male fertility possibly by the function of DNA repair. Mechanism not known. (Cheung et al., 2019)				
	Implantation		Transcriptional regulation on EMT-MET specific genes. Mechanism not known (Varghese et al., 2021)			
	Pluripotency and reprogramming		Loss in expression induce loss in the repressive MacroH2A.1 on the *Cdh1* promoter and incorporation of H3K4me2 mark at promoter of *Nanog*, *Klf4* for its activation (Syed et al., 2016)			
HIRA	Spermatogenesis		H3.3 incorporation in sperm chromatin during XY body formation (van der heijden et al., 2007; Szenker et al., 2011)			
	Oogenesis		H3.3 incorporation for transcriptional regulation (Nashun et al., 2015)	H3.3 incorporation for chromatin assembly (Ray-Gallet et al., 2002)		
	Fertilization	Male pronucleus formation (Smith R et al., 2021)	Replacement of protamine with H3.3 incorporation for male pronucleus formation (van der Heijden et al., 2005; Lin et al., 2014)		Along with yemanuclein, H3.3 incorporation in paternal chromatin in male pronucleus (Bonnefoy et al., 2007; Orsi et al., 2013)	
		Mechanism is not known	HIRA/CABIN/UBN1 regulates male pronucleus formation independent of H3.3 incorporation (Smith R et al., 2021)			
	Gastrulation		Cell cycle dependent transcriptional regulation. (Roberts et al., 2002)	H3.3 deposition (Szenker et al., 2012)		
	Neural differentiation		Recruitment of SETD1A for H3K4me3 deposition at *β-catenin* promoter (Li Y et al., 2017)		Yemanuclein associated H3.3 incorporation for regulation of intellectual disability gene (*dBRWD3*) (Chen et al., 2016)	
	Hematopoietic differentiation		H3.3 incorporation in the enhancer of RUNX1 (Majumder et al., 2015)			
			H3.3 deposition in the enhancer of troponin genes (Dilg et al., 2016)			
			EKLF/HIRA/H3.3/ASF1A complex regulates *β-globin* locus (Soni et al., 2014)			
	Muscular differentiation	Dephosphorylation of HIRA recruits H3.3 at *MYOD* gene for activation (Yang et al., 2016)	H3.3 recruitment by HIRA for *MyoD* activation (Yang et al., 2011)			
	Pluripotency and reprogramming	HIRA/Prohibitin complex deposits H3.3 on the Isocitrate dehydrogenase gene to regulate the self renewal of hESCs (Zhu et al., 2017)	HIRA mediated H3.3 deposition and enrichment of H3K27me3 on bivalent gene promoter negatively correlates with pluripotency (Banaszynski et al., 2013)			
SPT6	Muscular differentiation					SPT6 counteracts repressive mark H3K27me3 effect on *MyoD* gene activation (Wang et al., 2013)
	Pluripotency and reprogramming		Interacts with SUZ12 and counteracts with the repressive H3K27me3 enrichment at ESC super enhancers and promote pluripotency (Robert, 2017)			
ATRX	Oogenesis		ATRX maintains the H3 phosphorylation at the peri-centromeric heterochromatin region in MII oocyte for the chromatin condensation (Baumann et al., 2010)			
	Pre-implantation		Transmission of aneuploidy, mechanism not known (Baumann et al., 2010)			
	Muscular differentiation		Heterochromatin formation by recruiting H3.3 along with DAXX and lncRNA (Park et al., 2018)			
NAP1	Neural differentiation		NAP1L1 regulates SETD1A mediated H3K4me3 on *Rassf10* promoter for NPC differentiation (Qiao et al., 2018)			
			NAP1L2 recruits H3/H4 and establishes the H3K9/14 acetylation mark at the *Cdkn1c* promoter for neuronal differentiation (Attia et al., 2007)			
	Hematopoietic differentiation	NAP1L3 in umbilical cord blood derived HSCs induces G0 arrest of cell cycle and reguate the expression of E2F, MYC, HOXA3 and HOXA5 (Heshmati et al., 2018)		NAP1L is involved in transcriptional regulation of *α-Globin* expression and precursor genes for Erythroid differentiation (Abu-Dayaet al., 2005)		
	Pluripotency and reprogramming		NAP1L1 involved in nucleosome eviction at the time of ESC differentiation by complexing with FOXA2 coupled with SWI/SNF and INO80 and H2A.Z (Li et al., 2012)			
NPM	Oogenesis		NPM2 functions as a hub for the NLB proteins to assemble in GV oocyte (Inoue and Aoki, 2010)			
	Fertilization		NPM2 acts as a protamine acceptor for sperm chromatin decondensation in the NLB of fertilized oocyte (Inoue et al., 2011)	NPM2/H2A-H2B dimers get phosphorylated and decondenses the sperm chromatin (Platonova et al., 2011)		
CAF1	Spermatogenesis				CAF1 180/P75 replaces/associates with histones for protamines (Doyen et al., 2013; Doyen et al., 2015)	
	Oogenesis			CAF1 subunit, RBAP48, deacetylates histones, thereby the transcriptional silencing during chromatin assembly (veramaak et al., 1999)	CAF1 P180 recruits H3 for self-renewal of oogonia (Song et al., 2007; Anderson et al., 2011; Clémot et al., 2018)	
	Pre-implantation		Heterochromatin organization. Possible mechanism could be by H3.1/H3.2 deposition or recruiting (Mechanism is not known) (Houlard et al., 2006)	Replication dependent chromatin assembly by H3.1/H3.2 deposition in the proliferative cells of the embryo (Quivy et al., 2001)		
			Hypomethylated pre-implantation embryos are protected by enrichment of repressive marks H4K20me3, H3K9me3 (Hatanaka et al., 2015)			
	Gastrulation			Replication dependent chromatin assembly by H3.1/H3.2 deposition (Quivy et al., 2001)	CAF1- mediated PRC2 silencing regulates the senseless gene expression (Anderson et al., 2011)	
	Pluripotency and reprogramming		CAF1-PCNA establishes facultative heterochromatin by enriching H3K27me3 at pluripotency associated genes (Cheung et al., 2019)			
			2C like state upon CAF1 depletion brought about by the activation of retro elements and 2cell specific genes (Ishiuchi et al., 2015)			
ASF1	Spermatogenesis		Regulates the expression of STRA8 for mitotic to meiotic shift (Messiaen et al., 2016)			
	Fertilization				ASF1 transfer the H3.3 to HIRA for sperm chromatin decondensation (Horard et al., 2018)	
	Muscular differentiation		Recruits H3.3 along with HIRA on *MyoD* gene for its activation (Yang et al., 2011)			
	Pluripotency and reprogramming	In hESC, ASF1A regulates the expression of *OCT3/4, NANOG, SOX2* and *DNMT3B* by recruiting H3K56 acetylation mark at the respective promoters (Gonzalez-Muñozetal.,2014				
DAXX	Spermatogenesis		H3.3 incorporation in sperm chromatin for XY body formation (Rogers et al., 2004; Szenker et al., 2011)			
	Pre-implantation		H3.3 incorporation in pericentric heterochromatin in 2-cell embryos (Arakawa et al., 2015)			
	Muscular differentiation		Heterochromatin formation by recruiting H3.3 along with ATRX and lncRNA (Park et al., 2018)			

The detailed involvement of histone chaperones in development discussed in this review highlights the importance of these epigenetic factors quite vividly. However, a pronounced existing void is quite evident on the function of the histone chaperones in human development. But are there any future alternatives? Understanding development and its regulation at the epigenetic level are equally or rather more pertinent in humans. Unfortunately, till date, the model system of animals has been exploited to determine their role in human development. But, the extrapolation of findings from rodent-based studies will be a bottleneck to its implication in human development as physiological evidence indicates a significant difference in rodent vs. human development. Only a few reports involving *in vitro* studies could analyze how these chaperones might regulate stages of development in humans (as discussed in the review). But, almost nothing has been studied considering the actual development. We have discussed how ASF1A is indispensable for the generation of human iPSCs or CHAF1A loss induces a 100% increase in the generation of iPSCs. These reports stress the fact that how important these chaperones could be especially if we have to consider the clinical relevance for the generation of isogenic or allogenic iPSCs. Hence, the studies on the role of histone chaperones in human pre and post-implantation development should be undertaken to realize the importance of these epigenetic factors in designing clinical interventions. But, the hurdle to this is the complexity of using a human embryo as a model of development. As a breather, recent advances in the field of regenerative biology, scientist and clinicians are successfully exploiting the human organoids to study diseases, screen drugs among other usages (Kim et al., 2020). Organoids are generated from human pluripotent stem cells, both ESCs and iPSCs, and also from patient biopsy samples. Organoids represent organs in the dish generated from stem cells bestowed with the potential to self-organize in three-dimensional *in vitro* culture environment. Hence, human organoids recapitulating the *in vivo* organogenesis can be perfectly employed to study the role of histone chaperones. But, organoids could not serve as the pre-and post-implantation model for human development. Interestingly, that barrier could be overcome by the use of embryos being formed *in vitro* using the organoid models. Recently, blastocysts like structures, called blastoids have been developed from human embryonic stem cells and trophoblast stem cells that could replicate the phenomenon occurring within a blastocyst developed *in vivo* (Yanagida et al., 2021; Yu et al., 2021). The formation of gastruloids from ESCs further adds to the generation of the post-implantation model involving gastrulation (Moris et al., 2020; van den Brink et al., 2020). So, the function of the histone chaperones could be investigated in these models and a significant proportion of undiscovered mechanisms could be deciphered in the context of human development that could aid in the generation of better clinical interventions.

As discussed in the review, for somatic cell nuclear transfer and iPSC generation, epigenetically regulated chromatin accessibility becomes the barricade in nuclear reprogramming to preserve the cell identity. Hence, an understanding of the epigenetic regulators in cell fate determination and lineage specification is of utmost importance for unraveling the pathways and generation of patient-specific iPSCs or disease models.
